# Emotional Regulation as a Remedy for Teacher Burnout in Special Schools: Evaluating School Climate, Teacher’s Work-Life Balance and Children Behavior

**DOI:** 10.3389/fpsyg.2021.655850

**Published:** 2021-07-13

**Authors:** Sri Mulyani, Anas A. Salameh, Aan Komariah, Anton Timoshin, Nik Alif Amri Nik Hashim, R. Siti Pupu Fauziah, Mulyaningsih Mulyaningsih, Israr Ahmad, Sajid Mohy Ul din

**Affiliations:** ^1^Educational Administration, School of Postgraduate, Universitas Pendidikan Indonesia, Bandung, Indonesia; ^2^Department of Management Information Systems, College of Business Administration, Prince Sattam Bin Abdulaziz University, Al-Kharj, Saudi Arabia; ^3^Educational Administration, Universitas Pendidikan Indonesia, Bandung, Indonesia; ^4^Department of Propaedeutics of Dental Diseases, I.M. Sechenov First Moscow State Medical University, Moscow, Russia; ^5^Faculty of Hospitality, Tourism and Wellness, Universiti Malaysia Kelantan, Pengkalan Chepa, Malaysia; ^6^Manajemen Pendidikan Islam, Universitas Djuanda, Bogor, Indonesia; ^7^Public Administration, Postgraduate School, Universitas Garut, West Java, Indonesia; ^8^School of Business Management, Northern University of Malaysia, Sintok, Malaysia; ^9^Department of Management Science, University of Lahore, Gujrat, Pakistan

**Keywords:** teacher burnout, working conditions, work-life balance, children’s behavior, special schools, Pakistan

## Abstract

This research aimed to identify whether improvement in working conditions, children’s classroom behavior and work-life balance can lower teacher burnout ratio in Pakistan’s special schools by using techniques such as emotions regulation. The researcher employed a quantitative research methodology to fulfill the research’s purpose. The data for this research was collected using a questionnaire-based instrument. The confirmatory factor analysis and structural equation modeling techniques were used to test the construct validity and underlying structural relationships. The findings demonstrated that the impacts of all three variables are significant in reducing job burnout in teachers. Emotional regulation helps decrease the impact of working conditions and the children’s behavior. Nevertheless, it does not aid work-life balance as it requires other techniques of emotional regulation. The research is significant as it highlights the importance of overall working conditions’ improvement for teachers working with special needs children. The improvements are essential because the teachers must take extra effort and emotions into their job compared to a typical teacher. The researcher has highlighted the key finding, implications and limitations of this research besides suggesting directions for future research to facilitate peer researchers.

## Introduction

Teaching is a noble field as it shapes the younger generation to helm a nation in the future. Simultaneously, teachers suffer from multiple issues that take a toll on their emotional demands (Lee, 2019). Consequently, these demands result in negative workplace attitudes, such as burnout and reduced productivity (Akhtar Malik, 2019). The characteristics and teaching style in special schools are unique compared to teaching in regular schools. The teachers in special schools must professionally engage in management’s best practices and provide instructions due to the complexity of their roles and students (Wulandari and Djoehaeni, 2019; Ramdan et al., 2020). These teachers must assess the challenges posed by each student, advocate for them, communicate and collaborate with their parents.

The special school teachers are more prone to suffer from burnout than the general education teachers (Berry, 2011; Jovanović et al., 2019). These teachers are increasingly facing burnout due to critical issues as they have to maintain students’ discipline and the fear of verbal or physical abuse caused by the special students (Ramdan et al., 2020). The Pakistan government is focused on opening additional special education schools to cater to special children’s needs in all Pakistan provinces. There are 302 special schools in Punjab province that provide education to more than 35,000 special children. Nevertheless, there is a shortage in the supply of teachers. Thus, the currently employed teachers are facing more incidents of burnout. Hence, it is essential to help them relieve the stress through emotional regulations.

**FIGURE 1:**
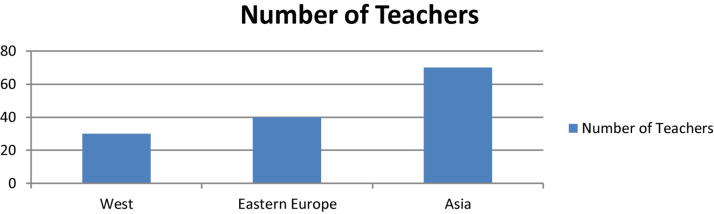
No. of teachers who face Burnout (region-wise). Reproduced from Ramdan et al. (2020) with permission.

This issue has resulted in a sustained decrease in special education teachers in the United States (Park and Shin, 2020). According to the Office of Post-secondary Education of the U.S. Department of Education Office of Postsecondary Education (2017), 46 states face a severe shortage of special education teachers. According to Park and Shin (2020), the teachers’ physical and psychological well-being, work performance and turnover are related to burnout. Burnout in teachers’ can impact teaching and the ways teachers interact with students in educational settings (Park and Shin, 2020). Moreover, Figure 1 show the number of teachers (region-wise) who faced Burnout level. The ratio of Asia is higher than other regions.

Teachers’ burnout develops over time by becoming persistent and causes deteriorating performance (Perrone et al., 2019). Therefore, research on special education teachers’ burnout is a significant concern and is reviewed extensively (Brunsting et al., 2014). Nonetheless, successful burnout prevention techniques do not exist (Hastings and Bham, 2003). Brunsting et al. (2014) has compiled data on special education teachers’ burnout from 1979 to 2013. The researchers examined 23 studies that assessed teacher burnout from the perspective of emotional fatigue and a sense of personal achievement. The findings demonstrated that stress management and emotional control imposed an impact on reducing burnout.

Furthermore, Brunsting et al. (2014) were the last to examine special education teacher burnout, and their research focused on the United States. Nevertheless, according to Park and Shin (2020), the problem of special education teacher burnout is gaining attention in countries such as the United Kingdom (UK), the Netherlands, Greece, Turkey, Iran, and the United States (Hastings and Bham, 2003; Zarafshan et al., 2013; Sarýçam and Sakýz, 2014; Hopman et al., 2018). The studies frequently reported higher burnout levels among special education teachers than general education teachers. Conversely, the generalized fact is derived from the studies that were conducted in other countries. Therefore, the researchers focused on the Pakistani special education system to extend the scope of the study.

The studies exploring the notion of burnout in the teaching profession is fragmented and insufficient despite special school teachers’ being more vulnerable to burnout. The majority of research has been conducted on teachers’ burnout in regular schools (Ramdan et al., 2020). Lee et al. (2016) and Lee (2019) highlighted the significance of emotional labor and emotional regulations among the teachers to demonstrate that they were facing burnout. Nevertheless, the literature on emotional regulation is limited.

Hence, the main objective of this study is to explore emotional regulation as a remedy for teacher burnout in special schools by considering the school climate, teachers’ work-life balance and children behavior in Pakistan to fill the gaps in the literature. Thus, the objectives of this study are:

• To investigate the impact of work climate on teacher burnout.
• To analyze the effect of work-life balance on teacher burnout.
• To examine the influence of children’s behavior on teachers’ burnout.
• To analyze how emotional regulation mediates these relationships.

This study has contributed to the theoretical knowledge by investigating the underlying mechanism in the relationship between work climate, work-life balance and children’s behavior with teacher burnout by exploring the role of emotional regulation. Additionally, this research also aids in enhancing workplace positivity by reducing job burden and increasing productivity. Policymakers and managements in Pakistan can benefit from the study’s findings by improving the policies, providing the best practices and giving proper training to the teachers. The study’s findings will also directly affect the rethinking process in challenging situations through emotional regulation to reduce job burden and improve children’s behavior.

Moreover, the study has implications for special schools and other educational institutions worldwide, including Pakistan. Teachers’ significant role is undeniable. They must be protected from facing burnout by assisting in emotional regulations when dealing with problems related to work climate, balancing work and life, and adapting to the children’s behavior for superior performance. This article started with an introduction and a detailed literature review on the variables in Section “Literature Review” that provide the basis for the illustrated research model. Subsequently, the research methodology is discussed in Section “Materials and Methods,” whereas Section “Analysis and Results” elaborates on the research results by providing empirical interpretation. Section “Discussion and Conclusion” includes discussion, conclusion, limitations and research implications of the study.

## Literature Review

### Supporting Theory

**FIGURE 2:**
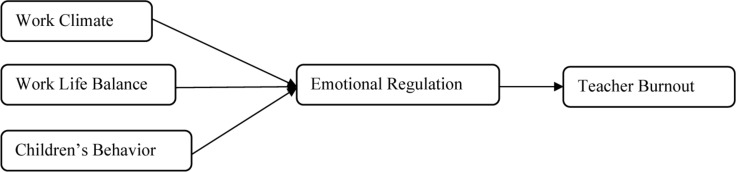
Research model.

The present study was undertaken based on the model of Job Demands-Resources. The model describes that workers suffer from exhaustion, job stress, and burnout when job demands are higher than the resources available to them (Demerouti et al., 2001; Lesener et al., 2019). This model is parallel with the theory of conservation resources, which postulates that inadequate or limited resources lead to burnout, resulting in decreased performance and negative job attitudes (Hobfoll et al., 2000). The model and the theory are specifically relevant for the teachers in the special education field as there is a chronic shortage of teachers forcing them to be vulnerable to burnout and prone to quitting jobs (Bettini et al., 2020). Figure 2 shows that research model for this study.

Hypothesis 1 (H1): Work climate has a significant relationship with teacher burnout.

### Work Climate and Teacher Burnout

Burnout can be referred to as the feeling of depersonalization owing to declining personal accomplishment and emotional exhaustion (Ramdan et al., 2020). A teacher’s job is stressful, whether in a private establishment, public school, kindergarten, primary education, secondary education, regular stream, or special education (Malinen and Savolainen, 2016). There are many job demands to be fulfilled (Goddard et al., 2006). The school climate can be defined as the psychological and social factors in which the teachers teach and fulfill their work duties (Malinen and Savolainen, 2016). A poor school climate and working culture can result in mental and physical exhaustion among the teachers leading to burnout (Fore et al., 2002; Grayson and Alvarez, 2008).

The aspects of school climate, such as the classroom, role conflict in teachers, external locus of control, peer support, and other aspects are critical. Teachers will not be satisfied with their work and may feel ambiguous about their work if these aspects are not favorable. In this situation, the teachers will not report their work environment positively (Dorman, 2003).

Previous studies reported that good working conditions, school structure, and professional interactions significantly improve the work climate and lead to further satisfaction among the teachers (Lavian, 2012; Garwood et al., 2018). In contrast, unsupportive administration and lack of recognition were considered prime factors contributing to burnout among the special education teachers (Perrone et al., 2019; Soini et al., 2019). Hence, this discussion implies that work climate can significantly predict burnout among teachers. Thus, the following hypothesis was made:

### Work-Life Balance and Teacher Burnout

According to Boström et al. (2020), it was found from a study on Sweden’s elementary school teachers that work-life balance can contribute to the teachers’ social well-being, and burnout can be reduced. Capone et al. (2019) stressed work-related factors, including balancing work and personal life. As per the Work-Life Model (WLM), work-life balance reduces depression and burnout among school teachers. Work stress is linked to work performance. The teachers’ performance is adversely affected if they cannot balance family and work (Pita, 2019).

Jang (2009) and Robinson et al. (2019) reported that special education school teachers are under stress. They must cope with numerous roles, such as managing, instructing, guiding, and other roles. This stress exerts a toll on their personal lives and can negatively disrupt their relationships. Hence, their performance at school can also be impacted since they may feel devalued for their expertise and work, leading to burnout. The findings imply that work-life balance has a significant association with teachers’ burnout. Hence, the following hypothesis was developed:

Hypothesis 2 (H2): Work-life balance has a significant relationship with teacher burnout.

### Children’s Behavior and Teacher Burnout

Nicholls et al. (2020) highlighted the correlation and prevalence of children’s challenging behavior in special schools’ settings. Besides, it has been proven that the teachers are exhausted and stressed out when they have to teach children with disorders such as motor skills, social behavior issues, and intellectually disabled children as compared to the regular children due to the challenges faced (Jovanović et al., 2019). Another study reported that the increasing levels of teacher burnout in special schools is linked to the challenging behaviors exhibited by the special children (Sun et al., 2019; Din et al., 2021).

Afreen (2021) have also reinforced similar results in their studies. Their studies found that the complex behavior of special children can pose difficulties for the children owing to their behavior management for those who suffer from disorders or autism (Hastings and Bham, 2003; Finlay et al., 2019; Ahmad and Ahmad, 2021). The findings prove that challenging children behavior can lead to teachers’ burnout in special schools. Hence, the following hypothesis was proposed:

Hypothesis 3 (H3): Children’s behavior can significantly affect teacher burnout.

### Mediating Role of Emotional Regulation

Burnout is stress faced by the workers. Stress is a type of unfavorable emotional experience that arises from environmental pressure. Consequently, there is a negative effect on the individual’s personality, and negative emotional reactions are displayed (Akhtar Malik, 2019). The workers can manage climate issues and other challenging aspects of the work variables, such as balancing family and work to control their emotions based on the situation and act accordingly. Hence, there will be lesser burnout on the teachers. Thus, there is a vital role played by managing emotions in the learning and teaching process (King and Chen, 2019). Previous studies have proved that effective management of emotions by utilizing emotional intelligence can reduce the burnout syndrome among the special education teachers (Puertas Molero et al., 2019; Obomeghie and Ugbomhe, 2021).

Secondary school teachers in Pakistan have shown improvement in their satisfaction level through regulating their emotions using emotional intelligence (Pervaiz et al., 2019; Nadeem et al., 2020). Lee (2019) and Park and Shin (2020) proved that physical education teachers’ effective regulation of emotions through emotional labor strategies could reduce burnout. Emotional regulation also changed their intention to quit the job. The review of previous literature shows that emotional regulation can serve as the underlying path in the relationships of work climate, work-life balance and children’s behavior with teachers’ burnout. Hence, the following hypotheses were developed:

Hypothesis 4a (H4a): Emotional regulation significantly mediates the relationship between work climate and teacher burnout.
Hypothesis 4b (H4b): Emotional regulation has a significant mediation effect on the association of work-life balance with teacher burnout.
Hypothesis 4c (H4c): Emotional regulation significantly mediates children’s behavior and teacher burnout.

## Materials and Methods

### Participants and Procedures

The participants of this study were teachers from the special education schools in Punjab, Pakistan. Punjab was chosen because it is the most populated province in Pakistan and Table 1 shows that 302 special education school in Punjab, Pakistan out of 420. Overall, 323 participants enrolled in this study. The sample was composed of 53% male (*n* = 169) and 47% female (*n* = 154). In terms of age group, most of the respondents were 41–50 years old, with 30% of the respondents. Secondly, 29% were from the age group of 31–40 years, whereas 25% of the total respondents were between 21 and 30 years. The respondents aged above 50 made up 15% of the respondent. According to respondents’ education levels, 43% were post-graduates, 33% were at master’s level, and 12% were at the graduate level, whereas another 12% had other qualifications.

**TABLE 1:** Number of special education schools in Pakistan (province wise).

	Number of special schools
Punjab	302
Sindh	75
KPK	39
Balochistan	4

The study conducted employed a quantitative methodology and a cross-sectional study. For this purpose, the researcher designed a survey instrument. The researcher employed a two-stage development and validation process to test the first draft of the survey questionnaire. Subsequently, the researcher contacted five professionals to examine the survey instrument’s validity. Three of the experts belonged to academia, whereas the other two were teaching specialists. The panel’s feedback was used to improve the survey’s quality by rephrasing and rearranging the item sequences for refining the clarity and readability.

The target population comprised of teachers working in Pakistan’s special schools, specifically from 302 special education schools in the Punjab province. The purposive sampling technique was applied in selecting the schools from different Punjab cities in Pakistan. First, the schools’ head teachers were approached individually. Their consent to participate in this research was obtained after the study’s purpose was explained to them. The developed questionnaires were distributed to the administrative heads of 100 special schools. Each school was sent 20 questionnaires, respectively, to be self-administered by the teachers. The email was employed as the mode of dispatch and data collection. The invitation email included details of the study’s purpose, scope and the ethical considerations adopted in the data collection and analysis. Subsequently, the researcher followed up the invitation emails with reminder phone calls to obtain a maximum response.

A total of 452 questionnaires were received after the 2 months waiting period. The researcher finalized 323 responses to be included in the analysis after removing redundant, incomplete or irrelevant responses. The researcher also investigated non-response bias issues by contacting 20 non-respondents through phone calls after the waiting period (Sheikh and Mattingly, 1981). The researcher inquired about the reasons for the teachers’ not participating through the phone conversations. The non-respondent teachers highlighted the lack of information and not understanding the survey’s various constructs as the reasons for not participating.

### Measures

The researcher utilized previous studies and available literature to identify the included constructs in the study’s research model. The questionnaire was divided into two sections. In the first section, the respondents’ demographic were questioned. The second section comprised a set of 30 items to capture relevant data against the constructs. The constructs and their sources are listed below.

• *Work Climate*

Work climate calculated the impact working conditions exert on the teacher’s burnout pattern. It was measured using five items adopted and restructured from the previous works (Marinette, 2018; Addimando, 2019). The questionnaire aimed at measuring working condition was developed explicitly for educational purpose. The scale’s reliability was over the recommended threshold of α = 0.84.

• *Work-Life Balance*

The second latent construct of work-life balance calculated the impact of work-life balance on the teacher’s burnout pattern. It was measured by adopting and rewording three items from previous research by Jang (2009). The response values ranged from a scale of 5 (strongly agree) to 1 (strongly disagree). Items were reverse coded to ensure higher scale values indicated an excellent work-life balance. The coefficient alpha of work-life balance for the current study measured by these three items was α = 0.78.

• *Children’s Behavior*

The impact of the students’ or children’s behavior toward the teachers on the teachers’ burnout pattern was calculated using seven items adopted and rephrased from previous research by Hastings and Bham (2003). The questions focused on the typical positive behavior pattern as observed by the teachers. The response valued ranged from scale 5 to scale 1 (strongly agree to disagree strongly). In the original report (Hastings and Bham, 2003), the reliability of these items was good (α = 0.83). The reliability score of the scale was over the threshold value of α = 0.80 for this study.

• *Emotions Regulation*

Emotional regulation is the model’s intervening endogenous variable that calculates the impact that emotions regulation techniques impose on the teacher’s burnout pattern. It was measured using ten items adopted and redrafted from previous researches (Chang, 2009; Castillo-Gualda et al., 2019). Emotion regulation is the study’s mediating variable that aids in assessing and improving the usage of strategies for emotions that effectively handle the significant effect of working climate, work-life balance, and children behavior. The scale’s reliability was over the recommended threshold of α = 0.82.

• *Teacher Burnout*

The bounded endogenous variable in this research model used to calculate teacher burnout patterns was measured using five items adapted from previous research by Jensen and Solheim (2020). The component of burnout appears to be the most relevant concerning the measures of job burden in the current study compared to work-related, emotional regulation, children behavior, and working conditions. The report’s original version has demonstrated good validity (Cronbach’s alpha of 0.87). The items were measured using a five-point Likert-type scale from ranging 5 (strongly agree) to 1 (strongly disagree). The current study’s items were valued at Cronbach’s alpha of 0.84.

### Structural Equation Modeling

Structural equation modeling (SEM) is a multivariate statistical technique that allows the modeling of several latent and empirical constructs concurrently (Byrne, 2016). Thus, SEM is typically chosen for simultaneously estimating the relationships patterns between variables in this analysis. A more valid and reliable measurement estimation can be obtained from the SEM as a path analysis method (Hair et al., 2017).

**FIGURE 3:**
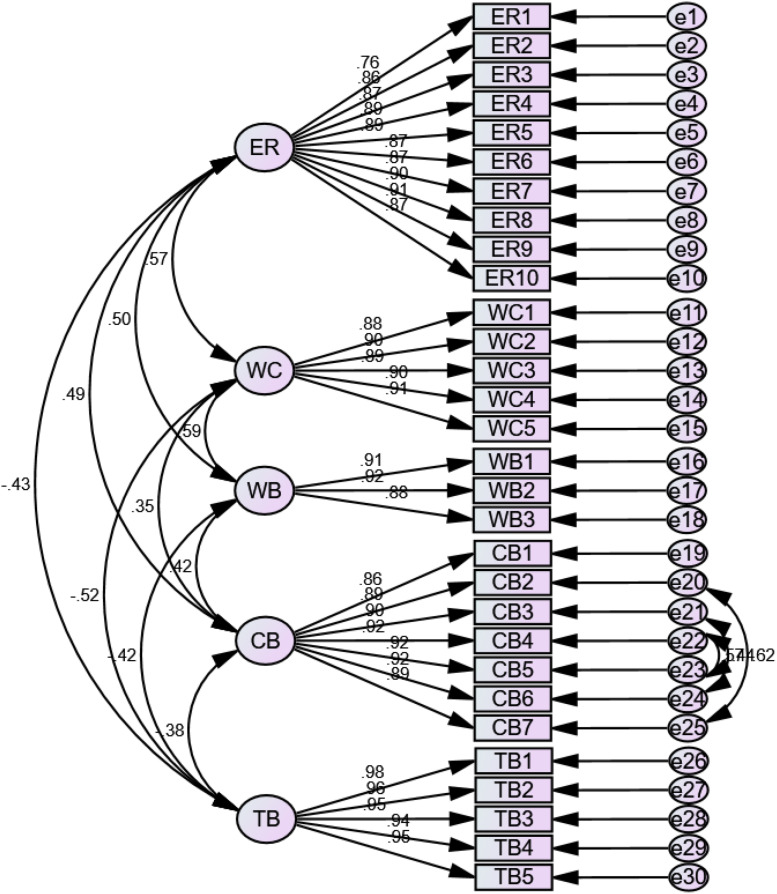
CFA measurement model.

**FIGURE 4:**
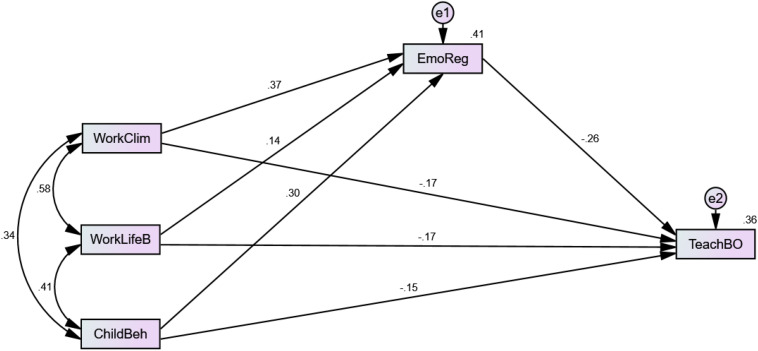
SEM measurement model.

In addition, SEM can be employed to demonstrate the total effects on both direct and indirect effects (Kline, 2015). SEM is the primary tool for hypotheses testing through a confirmatory approach rather than an exploratory approach resulting in mean regression analyses. Furthermore, the SEM technique can directly estimate measurement error rather than ignoring it, unlike other conventional methods (Hair et al., 2017). Analysis of covariance or causal modeling software (AMOS) 22.0 was used for the study’s analyses. In this research, Figures 3, 4 shows the CFA measurement model and SEM measurement model.

## Analysis and Results

The study’s descriptive statistics is demonstrated in Table 2. The results in this table show that no outliers are present in the data. Likewise, the values of the minimum and maximum statistics are within the bounds of the Likert scale used (scale 1 to 5).

**TABLE 2:** Descriptive statistics.

	N	Minimum	Maximum	Mean	Std. Deviation	Skewness	
	Statistic	Statistic	Statistic	Statistic	Statistic	Statistic	Std. Error
EmoReg	323	1.00	4.90	3.5440	1.10955	–0.806	0.136
WorkClim	323	1.00	5.00	3.4799	1.16093	–0.656	0.136
WorkLifeB	323	1.00	5.00	3.5492	1.10937	–0.765	0.136
ChildBeh	323	1.00	5.00	3.4430	1.10579	–0.611	0.136
TeachBO	323	1.00	5.00	2.4768	1.50553	0.759	0.136
Valid N (listwise)	323						

Moreover, the skewness results are within the threshold range of −1 to +1, indicating normality in the data. The Kaiser-Meyer-Olkin (KMO) test and Bartlett’s test were used to test the sample size and adequacy validity. The test’s results in Table 3 exhibits that the KMO statistic is 0.943, confirming the adequacy of the size.

**TABLE 3:** KMO and Bartlett’s test.

Kaiser-Meyer-Olkin measure of sampling adequacy		0.943
Bartlett’s test of sphericity	Approx. chi-square	12659.089
	df	435
	Sig.	0.000

Moreover, the Bartlett’s Test’s significance value shows acceptance of sphericity as it is below 0.05 value. The rooted component matrix is described in Table 4. Approximately the majority of the components carry a factor loading greater than 0.7. Thus, it is considered a good value, and no cross-loading issue was observed in any variable. ER1 has a value of 0.67 that is above the threshold 0.6 value.

**TABLE 4:** Rotated component matrix.

	Component				
	1	2	3	4	5
ER1	0.675				
ER2	0.770				
ER3	0.826				
ER4	0.856				
ER5	0.830				
ER6	0.840				
ER7	0.827				
ER8	0.845				
ER9	0.858				
ER10	0.843				
WC1				0.790	
WC2				0.827	
WC3				0.831	
WC4				0.846	
WC5				0.838	
WB1					0.804
WB2					0.832
WB3					0.868
CB1		0.830			
CB2		0.882			
CB3		0.884			
CB4		0.889			
CB5		0.895			
CB6		0.892			
CB7		0.877			
TB1			0.908		
TB2			0.907		
TB3			0.911		
TB4			0.904		
TB5			0.898		

Table 5 depicts the convergent and discriminant validity test results. Convergent validity is demonstrated through composite reliability (CR) values and average variance extracted (AVE) lower than the threshold values of 0.7 and 0.5. The values prove that the data has convergent validity. The results against the variables form a diagonal portion in the table, confirming that each variable is self-associated. The values are more significant than the initial values that confirm the discriminant validity.

**TABLE 5:** Convergent and discriminant validity.

	CR	AVE	MSV	CB	ER	WC	WB	TB
**CB**	0.968	0.810	0.244	**0.900**				
**ER**	0.969	0.756	0.321	0.494	**0.870**			
**WC**	0.954	0.806	0.345	0.349	0.567	**0.898**		
**WB**	0.929	0.814	0.345	0.419	0.498	0.587	**0.902**	
**TB**	0.921	0.711	0.266	–0.375	–0.429	–0.516	–0.418	**0.954**

*Bold values are represents the square root of AVE. Source, computed by research by using Amos.*

The indicators for confirmatory factor analysis are described the model of fitness from the statistical point of view. Hence, the model was fully supported. The model shows an excellent overlap between the indicators: minimum discrepancy per degree of freedom (CMIN/DF) = 2.319 ≤ 3, goodness of fit index (GFI) = 0.839 ≥ 0.80, incremental fit index (IFI) = 0.959 ≥ 0.90, comparative fit index (CFI) = 0.959 ≥ 0.90, and absolute fit index (RMSEA) = 0.064 ≤ 0.08.

The hypotheses testing were carried out using SEM. Table 6 shows that from the analysis of the first, the work climate variable was found to play a statistically insignificant direct effect on teachers’ burnout (*p* = −0.269^∗∗^, 17.2%). These findings highlighted that the working climate does not directly affect job burnout and will not solve this issue. Hence, H1 is not supported. Secondly, it was identified that work-life balance has a negative relationship with teacher burnout (*p* = −0.210^∗∗^, 17.4%). Thus, H2 is also not supported.

**TABLE 6:** Structural equation modeling.

Total Effect	ChildBeh	WorkLifeB	WorkClim	EmoReg
EmoReg	0.296**	0.138**	0.370**	0.000
TeachBO	−0.231**	−0.210**	−0.269**	−0.263**

**Direct Effect**	**ChildBeh**	**WorkLifeB**	**WorkClim**	**EmoReg**

EmoReg	0.296**	0.138**	0.370**	0.000
TeachBO	−0.153**	−0.174**	−0.172**	−0.263**

**Indirect Effect**	**ChildBeh**	**WorkLifeB**	**WorkClim**	**EmoReg**

EmoReg	0.000	0.000	0.000	0.000
TeachBO	−0.078**	−0.036	−0.097**	0.000

***significant value at 0.01, **significant value at 0.05, *significant value at 0.1, Source, Computed by Amos.

Similarly, the children’s behavior has no significant effect on the teacher burnout for H3 (*p* = −0.231^∗∗^, 15.3%). The results demonstrated a direct but statistically insignificant effect between the three independent variables and the dependent variable. Furthermore, according to H4a, the indirect effect of emotion regulation as a mediator variable is insignificant for work climate and teacher burnout. Hence, the findings did not support H4a.

Finally, the indirect effect of emotional regulation has a significantly positive mediation effect on the association of work-life balance with teacher burnout for the hypothesis H4b (*p* = 0.000, 7.8%). Similarly, emotional regulation has positively significant mediation for the relationship between children’s behavior and teachers’ burnout (*p* = 0.000, 9.7%) for hypothesis H4c. In accordance with the study’s results, the analysis suggested that emotional regulation supports the relationship of work-life balance and children’s behavior with teacher burnout. Emotional regulations aid teachers in becoming engaged, reduce their burden and employ various teaching practices. Thus, the results statistically provided evidence to support H4b and H4c.

## Discussion and Conclusion

### Discussion

The study aimed to investigate the role of work-life balance, work conditions and children behavior on the teachers’ burnout rate through the mediation role of emotions regulation among the teachers in Pakistan’s special. Three hypotheses were presented to test the direct impact, whereas additional three hypotheses were presented to test the indirect impact to fulfill the research’s objective. The first three hypotheses (direct paths) were not accepted and demonstrated a not reducing impact on the teachers’ burnout rate on the three independent variables without mediating variable.

The improvement of work conditions and work-life balance by singular units reduced the teacher’s burnout ratio by 17%, respectively. Besides, one unit’s improvement in the children’s behavior reduced the teacher’s burnout by 15% through emotional regulation. These results are supported by previous research with similar findings (Kanwar et al., 2009; Spence Laschinger et al., 2009; Shanafelt et al., 2012; Rubab, 2017).

Similarly, the researcher found that the impact of work climate did not improve by using emotional regulations techniques for the mediation impact. Antithetically, the impact of work life balance and children’s behavior improved through emotional regulation mediation by further reducing teacher’s burnout to 7.8 and 9.7%, respectively. These results are similar to findings of previous research (Tasca et al., 2009; Basim et al., 2013; Ju et al., 2015).

### Conclusion

The research was conducted in Pakistan specifically to assess the reasons for teacher burnout levels in special schools. The key findings of this study are given below:

• The teachers’ burnout rate can be reduced by improving the working conditions, such as pay rate, colleague behavior, and the school environment.
• The teachers’ burnout rate can be reduced if they can balance their work and personal life.
• The children’s behavior in the classroom can be a decisive factor for the teachers’ burnout rate. The teachers feel at ease, and the burnout rate is low if the children’s behavior is controlled and monitored.
• Strategies like emotion regulation can aid teachers in controlling the impact of work conditions on the burnout rate.

### Implications

The current study aimed to highlight multiple factors that impact teachers’ burnout patterns in Pakistan’s special schools. The study provides implications of theoretical nature by describing the variables in the context of Pakistan and highlighting the importance of emotional regulations for the teachers. In addition, the study’s finding had linked the development of emotional skills with capabilities to reduce the teachers’ burden from the practical implications. The development of the teaching emotional competencies is also linked to the study, considering that emotional skills management is a core demand for the teachers.

Implementing training programs focusing on the emotional management skills in work-life balance and managing job burden can support the teachers in reducing their job burden and enhancing well-being. Moreover, this study is expected to assist the policymakers of educational institutes for special children to develop regulatory policies that reduce teachers’ burnout rate and improve teachers’ training to assist them in learning emotion regulation techniques.

### Limitations and Future Research Recommendations

The research quality should be continuously improved by removing the various limitations and boundaries of a study. The first limitation of this study is the small sample size of the data collected. In addition, the study was conducted within Pakistan by focusing on the teachers working in special schools. The tests and approaches employed in this research were minimal. Hence, bearing the current study’s limitation, there is a scope for future researchers to increase the sample and population sizes in future studies.

Likewise, future researchers can employ other forms of tests and techniques to analyze the data. The data sample can be enlarged by collecting data from other schools or obtaining data from other countries. Moreover, the spectrum of this research is limited by the use of limited variables in this study. Because the study have mixed results so that Future researchers can widen the research spectrum by adding additional variables, such as the teachers’ mental state and the work satisfaction levels.

## Data Availability Statement

The original contributions presented in the study are included in the article/supplementary material, further inquiries can be directed to the corresponding author.

## Ethics Statement

Ethical review and approval was not required for the study on human participants in accordance with the local legislation and institutional requirements. Written informed consent to participate in this study was provided by the participants’ legal guardian/next of kin.

## Author Contributions

SM: conceptualization, project administration, writing, and review. AS: data curation, review, and editing. AK: methodology, supervision, and writing. AT and NH: data curation, methodology, supervision, and writing. RF and MM: formal analysis, review, and editing. SU: proofreading, editing, review, and analysis. IA: conceptualization, data analysis, project administration, writing, and review.

## Conflict of Interest

The author declares that the research was conducted in the absence of any commercial or financial relationships that could be construed as a potential conflict of interest.
